# Supplementary material for: Proteome Profile Changes Induced by Heterologous Overexpression of Mycobacterium tuberculosis-Derived Antigens PstS-1 (Rv0934) and Ag85B (Rv1886c) in Mycobacterium microti
Source: Biomolecules. 2022 Dec 8;12(12):1836. doi: 10.3390/biom12121836 (PMC9775975; doi:10.3390/biom12121836)
Supplement: Supplementary file 1 [file biomolecules-12-01836-s001.zip › biomolecules-1941840-supplementary.pdf]

A)

```
CLUSTAL O (1.2.4) multiple sequence alignment

Rv0934 (PstS-1)

M. microti          MKIRLHTLLAVLTAAPLLLAAAGCGSKPPSGSPETGAGAGTVATTPASSPVTLAETGSTL     60
M. tuberculosis     MKIRLHTLLAVLTAAPLLLAAAGCGSKPPSGSPETGAGAGTVATTPASSPVTLAETGSTL     60
                    ************************************************************

M. microti          LYPLFNLWGPAFHERYPNVTITAQGTGSGAGIAQAAAGTVNIGASDAYLSEGDMAAHKGL     120
M. tuberculosis     LYPLFNLWGPAFHERYPNVTITAQGTGSGAGIAQAAAGTVNIGASDAYLSEGDMAAHKGL     120
                    ************************************************************

M. microti          MNIALAISAQQVNYNLPGVSEHLKLNGKVLAAMYQGTIKTWDDPQIAALNPGVNLPGTAV     180
M. tuberculosis     MNIALAISAQQVNYNLPGVSEHLKLNGKVLAAMYQGTIKTWDDPQIAALNPGVNLPGTAV     180
                    ************************************************************

M. microti          VPLHRSDGSGDTFLFTQYLSKQDPEGWGKSPGFGTTVDFPAVPGALGENGNGGMVTGCAE     240
M. tuberculosis     VPLHRSDGSGDTFLFTQYLSKQDPEGWGKSPGFGTTVDFPAVPGALGENGNGGMVTGCAE     240
                    ************************************************************

M. microti          TPGCVAYIGISFLDQASQRGLGEAQLGNSSGNFLLPDAQSIQAAAAGFASKTPANQAISM     300
M. tuberculosis     TPGCVAYIGISFLDQASQRGLGEAQLGNSSGNFLLPDAQSIQAAAAGFASKTPANQAISM     300
                    ************************************************************

M. microti          IDGPAPDGYPIINYEYAIVNNRQKDAATAQTLQAFLHWAITDGNKASFLDQAHFQPLPPA     360
M. tuberculosis     IDGPAPDGYPIINYEYAIVNNRQKDAATAQTLQAFLHWAITDGNKASFLDQVHFQPLPPA     360
                    ***************************************************.********

M. microti          VVKLSDALIATISS*   374
M. tuberculosis     VVKLSDALIATISS*   374
                    ***************
```

B)

```
CLUSTAL O (1.2.4) multiple sequence alignment

Rv1886c (Ag85B)

M. microti           MTDVSRKIRAWGRRLMIGTAAAVVLPGLVGLAGGAATAGAFSRPGLPVEYLQVPSPSMGR    60
M. tuberculosis      MTDVSRKIRAWGRRLMIGTAAAVVLPGLVGLAGGAATAGAFSRPGLPVEYLQVPSPSMGR    60
                     ************************************************************

M. microti           DIKVQFQSGGNNSPAVYLLDGLRAQDDYNGWDINTPAFEWYYQSGLSIVMPVGGQSSFYS    120
M. tuberculosis      DIKVQFQSGGNNSPAVYLLDGLRAQDDYNGWDINTPAFEWYYQSGLSIVMPVGGQSSFYS    120
                     ************************************************************

M. microti           DWYSPACGKAGCQTYKWETFLTSELPQWLSANRAVKPTGSAAIGLSMAGSSAMILAAYHP    180
M. tuberculosis      DWYSPACGKAGCQTYKWETFLTSELPQWLSANRAVKPTGSAAIGLSMAGSSAMILAAYHP    180
                     ************************************************************

M. microti           QQFIYAGSLSALLDPSQGMGPSLIGLAMGDAGGYKAADMWGPSSDPAWERNDPTQQIPKL    240
M. tuberculosis      QQFIYAGSLSALLDPSQGMGPSLIGLAMGDAGGYKAADMWGPSSDPAWERNDPTQQIPKL    240
                     ************************************************************

M. microti           VANNTRLWVYCGNGTPNELGGANIPAEFLENFVRSSNLKFQDAYNAAGGHNAVFNFPPNG    300
M. tuberculosis      VANNTRLWVYCGNGTPNELGGANIPAEFLENFVRSSNLKFQDAYNAAGGHNAVFNFPPNG    300
                     ************************************************************

M. microti           THSWEYWGAQLNAMKGDLQSSLGAG* 325
M. tuberculosis      THSWEYWGAQLNAMKGDLQSSLGAG* 325
                     **************************
```

Figure S1. Protein sequence translation and alignments of genes A) Rv0934 and B) Rv1886c from strains *M. microti* and *M. tuberculosis* used in this study.

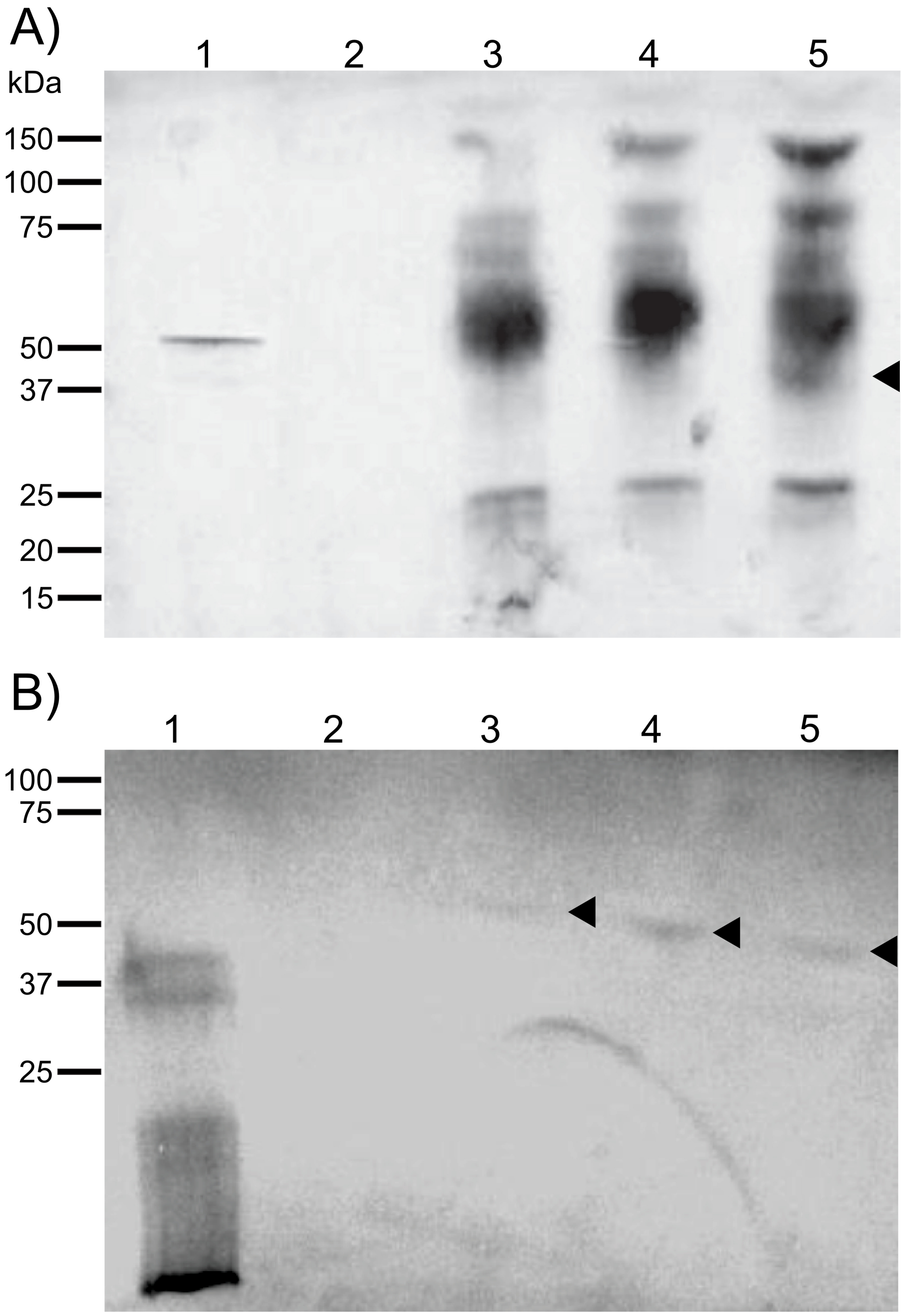

Figure S2. Differential expression and glycosylation of proteins from wild-type and recombinant *M. microti* strains. A) Mannose lectin blot, B) Sialic acid lectin blot.

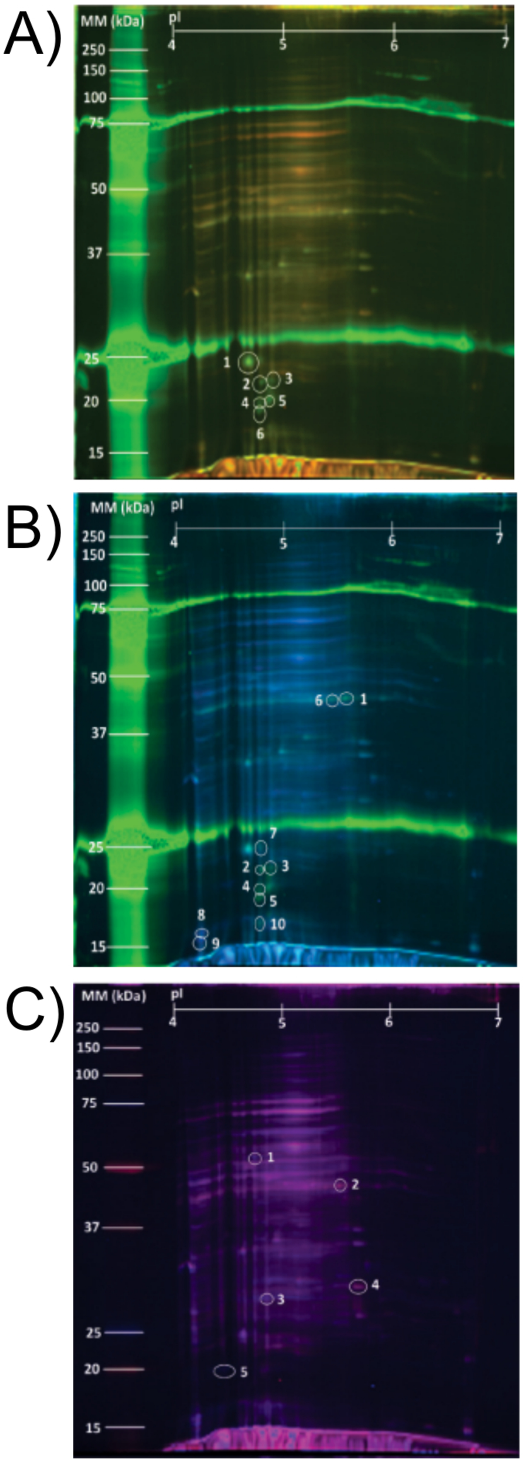

Figure S3. Two-dimensional fluorescence difference gel electrophoresis with labelled proteins from the cell extract of A) *M. microti* vs. *M. microti*-PstS-1, B) *M. microti* vs. *M. microti*-AG85B and C) *M. microti*-PstS-1 vs. *M. microti*-Ag85B.
